# Improved Stability and Practicality for Synthesis of 4-Borono-2-[^18^F]fluoro-l-phenylalanine by Combination of [^18^O]O_2_ Single-Use and [^18^F]CH_3_COOF Labeling Agents

**DOI:** 10.1007/s13139-021-00719-1

**Published:** 2022-02-08

**Authors:** Sadahiro Naka, Toshimitsu Watanabe, Yasukazu Kanai, Tadashi Watabe, Mitsuaki Tatsumi, Hiroki Kato, Eku Shimosegawa, Jun Hatazawa

**Affiliations:** 1 Department of Nuclear Medicine and Tracer Kinetics, Graduate School of Medicine, Osaka University, 2-2, Yamadaoka, Suita, Osaka 565-0871 Japan; 2 Department of Radiology, Osaka University Hospital, 2-15, Yamadaoka, Suita, Osaka 565-0871 Japan; 3 Radiochemistry and Targetry Section, Engineering Department, Medical & Advanced Equipment Unit, Industrial Equipment Division, Sumitomo Heavy Industries, 5-2, Soubiraki-cho, Niihama, Ehime 792-8588 Japan; 4 Department of Biofunctional Analysis, Faculty of Pharmacy, Osaka Medical and Pharmaceutical University, 4-20-1, Nasahara, Takatsuki, Osaka 569-1094 Japan; 5 Department of Quantum Cancer Therapy, Research Center for Nuclear Physics, Osaka University, 10-1, Mihogaoka, Osaka, Ibaraki 567-0047 Japan; 6 Department of Molecular Imaging in Medicine, Graduate School of Medicine, Osaka University, 2-2, Yamadaoka, Suita, Osaka 565-0871 Japan

**Keywords:** [^18^O]O_2_ gas, Single-use, [^18^F]F_2_, [^18^F]CH_3_COOF, [^18^F]FBPA, Boron neutron capture therapy

## Abstract

**Purpose:**

4-Borono-2-[^18^F]fluoro-l-phenylalanine ([^18^F]FBPA) synthesized with [^18^F]F_2_, produced using the ^18^O(p, n)^18^F reaction, has been reported for increasing radioactivity. However, a dedicated system and complex procedure is required to reuse the costly [^18^O]O_2_ gas; also, the use of [^18^F]F_2_ as a labeling agent reduces the labeling rate and radiochemical purity. We developed a stable and practical method for [^18^F]FBPA synthesis by combining [^18^F]F_2_, produced using a [^18^O]O_2_ single-use system, and a [^18^F]CH_3_COOF labeling agent.

**Methods:**

The produced [^18^F]F_2_ was optimized, and then [^18^F]FBPA was synthesized. For passivation of the target box, 0.5% F_2_ was pre-irradiated in argon. Gaseous products were discarded; the target box was filled with [^18^O]O_2_ gas, and then irradiated (first irradiation). Then, the [^18^O]O_2_ gas was discarded, 0.05–0.08% F_2_ in argon was fed into the target box, and it was again irradiated (second irradiation). The [^18^F]F_2_ obtained after this was passed through a CH_3_COONa column, converting it into the [^18^F]CH_3_COOF labeling agent, which was then used for [^18^F]FBPA synthesis.

**Results:**

The mean amount of as-obtained [^18^F]F_2_ was 55.0 ± 3.3 GBq and that of as-obtained [^18^F]CH_3_COOF was 21.6 ± 1.4 GBq after the bombardment. The radioactivity and the radiochemical yield based on [^18^F]F_2_ of [^18^F]FBPA were 4.72 ± 0.34 GBq and 12.2 ± 0.1%, respectively. The radiochemical purity and molar activity were 99.3 ± 0.1% and 231 ± 22 GBq/mmol, respectively.

**Conclusion:**

We developed a method for [^18^F]FBPA production, which is more stable and practical compared with the method using [^18^O]O_2_ gas-recycling and [^18^F]F_2_ labeling agent.

## Introduction

Boron neutron capture therapy (BNCT) is an anti-cancer treatment that is based on the ^10^B(n, α)^7^Li reaction in tumors, and the high linear energy transfer of the α and ^7^Li particles generated upon the irradiation of ^10^B [1–4]. Recently, the BNCT system was approved as a medical device for neutron irradiation, and ^10^B-4-borono-l-phenylalanine (^10^BPA) was approved as a ^10^B-carrier-drug for cancer cells, by the Ministry of Health, Labour and Welfare in Japan. Before neutron irradiation, the amount of ^10^B present in tumors and the surrounding normal tissues was typically evaluated using positron emission tomography (PET). Subsequently, 4-borono-2-[^18^F]fluoro-l-phenylalanine ([^18^F]FBPA) [5–8] was chosen because the distribution of [^18^F]FBPA in a tracer dose was correlated with that of a therapeutic dose of ^10^BPA in both rats [9, 10] and humans [7]. The availability of [^18^F]FBPA is critical to the successful determination of the cellular ^10^B levels to ensure treatment efficacy, and several approaches have been reported to address its synthesis. The [^18^F]FBPA synthesis developed by Ishiwata et al. [11] involves an electrophilic substitution reaction using [^18^F]CH_3_COOF or [^18^F]F_2_ for the direct ^18^F-labeling of the aromatic ring. In their process, Ishiwata et al. employed the ^20^Ne(d, α)^18^F nuclear reaction involving a small cross-section. By employing ^20^Ne(d, α)^18^F as the nuclear reaction, and [^18^F]CH_3_COOF as the labeling agent, the amount of radioactivity of the generated [^18^F]FBPA was 1200 ± 160 MBq by irradiation for 120 min [12]. When using an alternate method, [^18^F]F_2_ gas was produced by using two-step proton beam irradiation and the nuclear reaction of ^18^O(p, n)^18^F with [^18^O]O_2_ gas [13, 14]. The use of this method allowed the production of large amounts of [^18^F]F_2_ (102 ± 27 GBq) and [^18^F]FBPA (5.3 ± 1.2 GBq) [15], though it required a dedicated recycling system to recover the costly [^18^O]O_2_ gas and a complicated procedure [13, 14]. In addition, [^18^F]F_2_ was used as a labeling agent in a previous study [15]; however, it was reported that the direct electrophilic substitution of aromatic ring in BPA with extremely reactive [^18^F]F_2_ resulted in a lower ^18^F-labeling rate and radiochemical purity in [^18^F]FBPA compared with that with [^18^F]CH_3_COOF [11, 12].

The purpose of this study was to combine the advantages of these two methods and establish a more practical and stable synthesis process. That is, it involved the development of an efficient and economical ^18^O(p, n)^18^F reaction-based [^18^F]F_2_ production system that does not require any [^18^O]O_2_ gas-recycling and complicated procedures. The established method in this study involved the use of [^18^F]CH_3_COOF as the labeling agent for the stable synthesis of large amount of [^18^F]FBPA for clinical use.

## Material and Methods

### Cyclotron and Target Material

An energy proton beam of 18 MeV was obtained using CYPRIS-HM-18 (Sumitomo Heavy Industries, Tokyo, Japan). An aluminum (Al) target, originally used for the production of [^11^C]CO_2_, was employed as is for the [^18^F]F_2_ production. This target was conical in shape with a length of 154 mm, a front and back diameter of 20 and 30 mm, respectively, and a volume of 75 mL. The 18-MeV proton beam was decelerated to 14.4 MeV by passing it through a vacuum foil made of 10 μm thickness Havar and a target foil made of 600 μm thickness aluminum. Enriched oxygen-[^18^O]O_2_ gas (>98 atom%) was used as the target gas. Argon gas (>99.99995% pure) and argon gas mixed with 2% F_2_ gas were used for the passivation and recovery of the adsorbed [^18^F]F_2_ gas, respectively (Taiyo Nippon Sanso Corporation, Tokyo, Japan).

### Target System

**Fig. 1:**
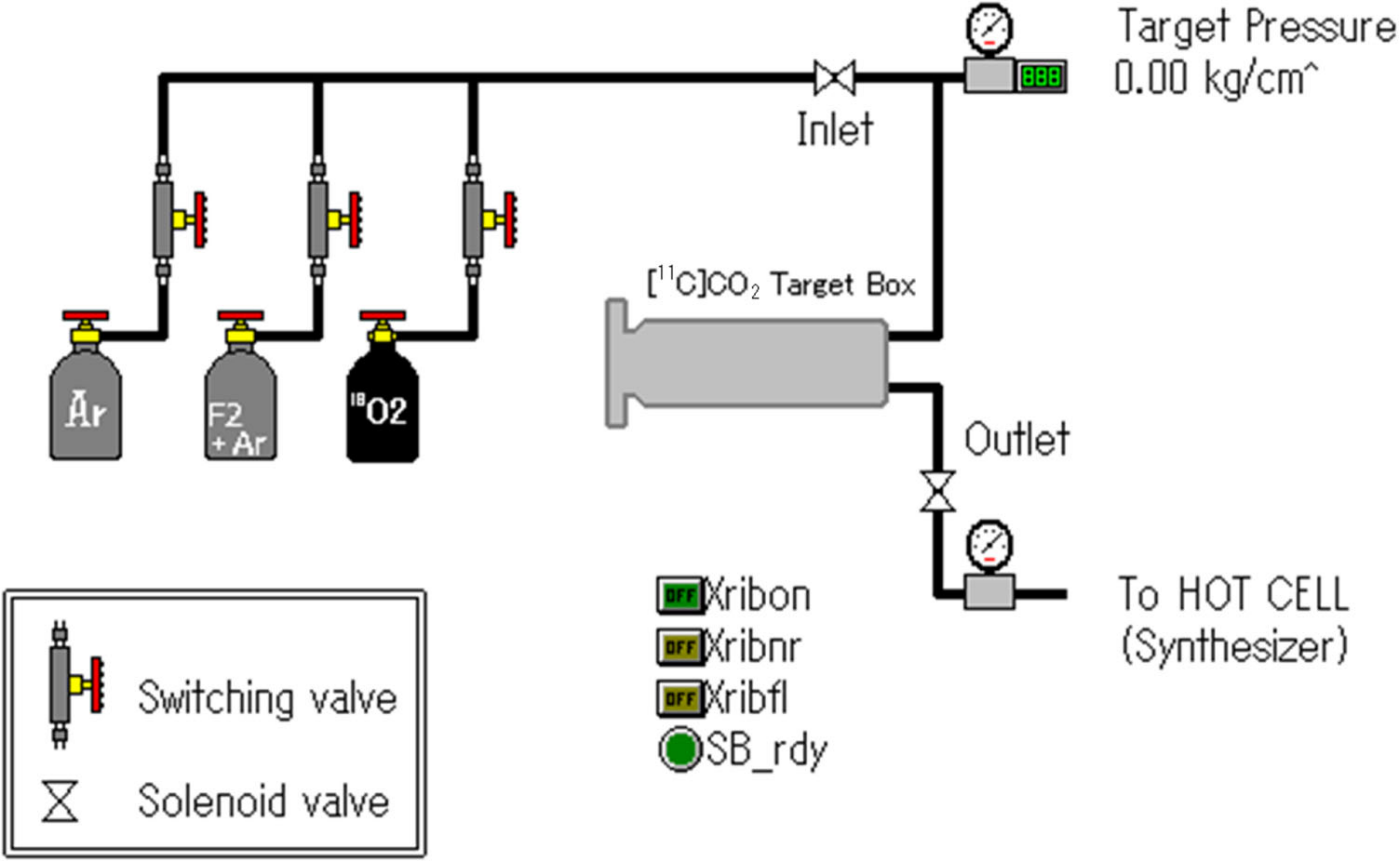
Schematic representation of target system with single-use of [^18^O]O_2_ gas for [^18^F]F_2_ gas production; valve-switching alone is performed manually while all other systems are controlled by cupid system

The target box was connected via three gas supply lines to the gas cylinders, with each line incorporating switching valves (Fig. 1). The three gas cylinders contained (1) [^18^O]O_2_ gas, (2) argon gas mixed with 2% F_2_ gas for target inner wall passivation or for the collection of the adsorbed [^18^F]F_2_ after [^18^O]O_2_ irradiation, and (3) argon gas for 2% F_2_ gas dilution and target system purging. The stainless-steel piping of the inlet and outlet lines, solenoid valves, and pressure gauges were used to construct this system. The valves were switched manually. The filling gas pressure, irradiation, and gas transfer to the synthesizer were controlled using an automated synthesis control system (Cupid system, Sumitomo Heavy Industries, Tokyo, Japan).

### Pre-irradiation of Target for Passivation

In this study, we employed a two-step irradiation method for the production of [^18^F]F_2_ [13, 14]. First, pre-irradiation was carried out for 10 min to passivate the surface of the target box with 0.5% fluorine in argon at a set pressure of 14.8 kg/cm^2^ and a proton beam of 17 μA. This operation is important not only for the passivation of the Al target but also for removing any contamination from the target [14, 16, 17]. Gaseous products from the pre-irradiation sequence were passed through a synthesizer installed in a hot-cell and then discarded. After the argon purge, the target pressure was reduced to atmospheric pressure.

### Confirmation of [^18^F]F_2_ Amount at Each Irradiation Step After Passivation

After pre-irradiation with 0.5% F_2_ mixed with argon, the first irradiation with [^18^O]O_2_ gas at 25 μA for 10 min and the second irradiation at 17 μA for 10 min with 0.03–0.9% F_2_ mixed with argon were carried out. The second irradiation was repeated twice (total three times), which were the third and fourth irradiations in the sequence according to the calculation of the total amount of [^18^F]F_2_ in the target box. The radioactivity of the gaseous product was preserved using a charcoal and soda-lime column attached to the waste line and both columns were measured at the same time using a dose-calibrator. The recovery rate of [^18^F]F_2_ from the target in each irradiation step was calculated as the sum of the radioactivity obtained at each irradiation step.

### Optimization of Proton Beam Current, Irradiation Time, and Fluorine Gas Concentration

A target box with a larger volume than that used in previous studies for [^18^F]F_2_ production [14, 16, 17] was selected; therefore, the irradiation conditions and F_2_ gas concentration for the recovery of [^18^F]F_2_ were optimized accordingly. During the second irradiation, the proton beam irradiation for 10 min at 5 μA or 17 μA and for 2, 10, or 20 min at 17 μA was used for the irradiation of argon with 0.05–0.08% F_2_ at 14.8 kg/cm^2^. In addition, variations in the F_2_ gas concentration in the 0–0.9% range (amounts in the range 0–448 μmol) were also investigated by irradiation at 17 μA for 10 min.

### Production of [^18^F]F_2_ Gas for [^18^F]FBPA Synthesis

After the pre-irradiation, the target box was filled with [^18^O]O_2_ gas at a pressure of 15.0 kg/cm^2^ and then irradiated with a proton beam current of 30 μA for 150 min (first irradiation). The resulting initially irradiated [^18^O]O_2_ gas was discarded via the same route used during the pre-irradiation step without any recycling. The target box was then filled with a 0.05–0.08% mixture of F_2_ gas in argon at a pressure of 14.8 kg/cm^2^ and was irradiated at a proton beam current of 17 μA for 20 min (second irradiation). Thereafter, the irradiated gas was collected and used to synthesize [^18^F]FBPA.

### Synthesis of [^18^F]FBPA Solution

**Fig. 2:**
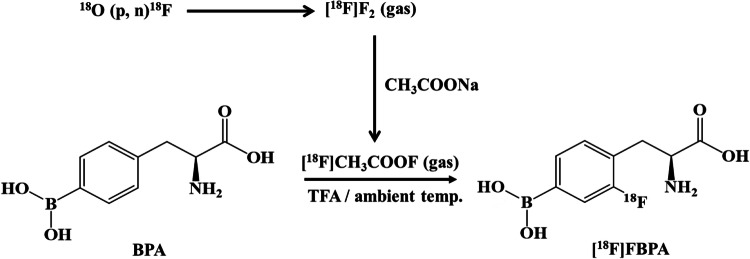
[^18^F]FBPA synthesis scheme; after conversion to [^18^F]CH_3_COOF using the CH_3_COONa column, [^18^F]FBPA was synthesized and allowed to react with [^18^F]CH_3_COOF and BPA in TFA solution at ambient temperature

The [^18^F]F_2_-containing gas obtained after the second irradiation was passed through a CH_3_COONa column, resulting in the conversion of [^18^F]F_2_ to [^18^F]CH_3_COOF and introduced into the reactor at a constant flow rate of 300 mL/min. The [^18^F]FBPA was synthesized with a cassette-type synthesizer CFN-MPS200 (Sumitomo Heavy Industries, Tokyo, Japan), as described in previous reports [11, 12]. The [^18^F]CH_3_COOF was introduced into a solution of 30 mg of 4-borono-l-phenylalanine (>97%, Matrix Scientific, Columbia, USA) dissolved in 4 mL of trifluoroacetic acid (TFA) at room temperature (Fig. 2). Subsequently, TFA was removed from the reactor under reduced pressure, while maintaining a N_2_ gas flow rate of 200 mL/min at 120 °C. The resulting residue was dissolved in 2 mL of 0.1% aqueous acetic acid and injected into a high-performance liquid chromatography (HPLC) column (YMC-Pack ODS-A 20 mm × 150 mm HPLC column (YMC, Kyoto, Japan)). The fraction containing [^18^F]FBPA was eluted at a retention time of approximately 17 min. The HPLC conditions were as follows: eluent at 0.1% aqueous acetic acid, flow rate at 10 mL/min, and ultraviolet (UV) detection at 280 nm, and a radioactivity detector was used. After drying the [^18^F]FBPA fraction with an evaporation, the residue was re-dissolved with approximately 14 mL of saline, and then, filter sterilization of the [^18^F]FBPA solution was carried out by passing it through a vented Milex-GS filter (Merck Millipore, Darmstadt, Germany). The filtered [^18^F]FBPA solution was collected in a 20 mL sterile vial and then added to a 25% ascorbic acid injection (0.2 mL) (Fuso Pharmaceutical Industries, Osaka, Japan) for pH adjustment.

### Quality Control for [^18^F]FBPA Solution

The quality of the [^18^F]FBPA solution was evaluated according to the criteria of the Short-lived Radiopharmaceutical Safety Management Committee of Osaka University Hospital. The criteria used for the evaluation were in accordance with the Standards of Compounds Labeled with Positron Emitting Radionuclides Approved as Established Techniques for Medical Use (2009 revision) [18], and were as follows: volume per batch, radioactivity, half-life, appearance (color and particles), endotoxin levels, sterility, pH, radionuclidic identity, radionuclidic purity, radiochemical purity, and residual solvent amounts (ethanol, acetic acid, and TFA). For the carrier amount of FBPA, the maximum dose per patient was set to <5 mg which is sufficiently safe, based on the doses in precious reports [19], and the administrable injection volume was calculated. HPLC for analysis was performed with YMC-Pack ODS AQ 4.6 mm × 150.0 mm column (YMC, Kyoto, Japan) and 50 mmol/L NaH_2_PO_4_ solution as eluent. The radiochemical purity and carrier amount of FBPA were measured using a radioactivity detector and UV detector at 280 nm, respectively, with a flow rate of 1.5 mL/min. The FBPA standard used for the measurement of the carrier amount was provided by Osaka Prefecture University. In the residual solvent test, acetic acid and TFA were used under the same conditions as the carrier amount of FBPA was measured at UV 210 nm and a flow rate of 0.5 mL/min. Residual ethanol was measured using gas chromatography with TSG-1 15% SHINCARBON A 60/80 (3.1 m × 3.2 mm I.D., Shimadzu, Kyoto, Japan). The injection port, column, and flame ionization detector temperature were set to 180 °C, 90 °C, and 180 °C, respectively. The carrier gas was nitrogen, while the flow rate was maintained at 30 mL/min. The pH value was determined by potentiometry using a F-72 pH/ion meter calibrated with a standard pH solution (Horiba, Kyoto, Japan), and the endotoxin test was carried out using a Toxinometer® ET-6000 (FUJIFILM Wako Pure Chemical). In addition, the enantiomeric purity of [^18^F]FBPA was evaluated by chiral HPLC with a Crownpak CR (-) 4.0 mm × 150 mm column (Daicel, Tokyo, Japan) and a perchloric acid aqueous solution (pH 2.0) at 1.0 mL/min at 25 °C.

## Results

**Fig. 3:**
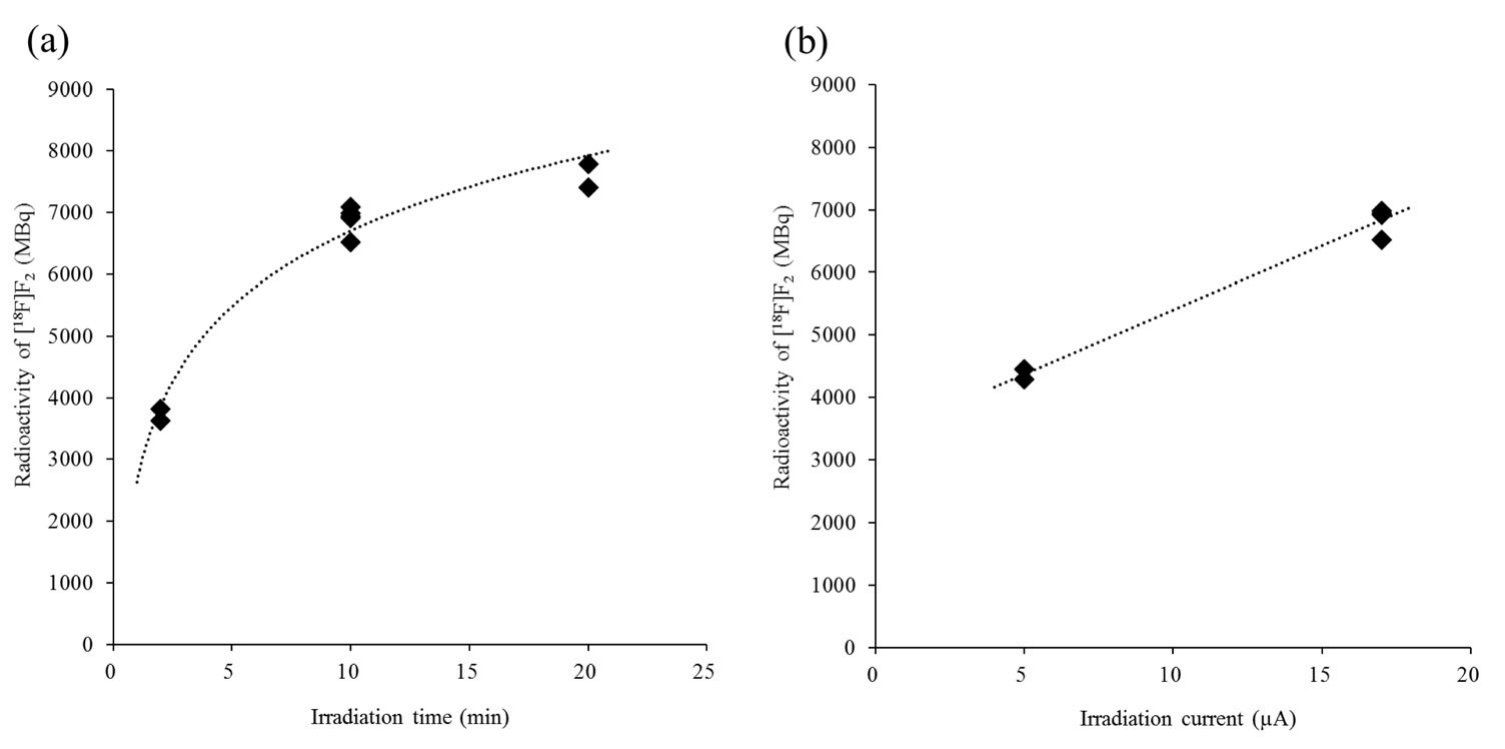
Radioactivity of [^18^F]F_2_ under each condition: **a** second irradiation time at 17 μA for 2 (*n*=2), 10 (*n=*5), and 20 min (*n*=2). Radioactivity was 3723 ± 132, 6891 ± 218, and 7598 ± 276 MBq, respectively; **b** beam current of cyclotron was set to 5 (*n*=2) or 17 μA (*n*=5) for 10 min. Radioactivity was 4369 ± 116, 6891 ± 218 MBq, respectively. Radioactivity (decay-correct to EOB) of [^18^F]F_2_ was depicted after second irradiation with 0.05–0.08% fluorine in target gas.

After the surface passivation in the target box with pre-irradiation, the recovery rate of [^18^F]F_2_ after the first, second, third, and fourth irradiations was 3%, 75%, 16%, and 6%, respectively, calculated from the total radioactivity. Figure 3 depicts the relationship between the radioactivity and proton irradiation time as well as that between the radioactivity and proton beam current in the second irradiation. The radioactivity of the collected [^18^F]F_2_ increased with increase in the proton irradiation time and the proton beam current.

**Fig. 4:**
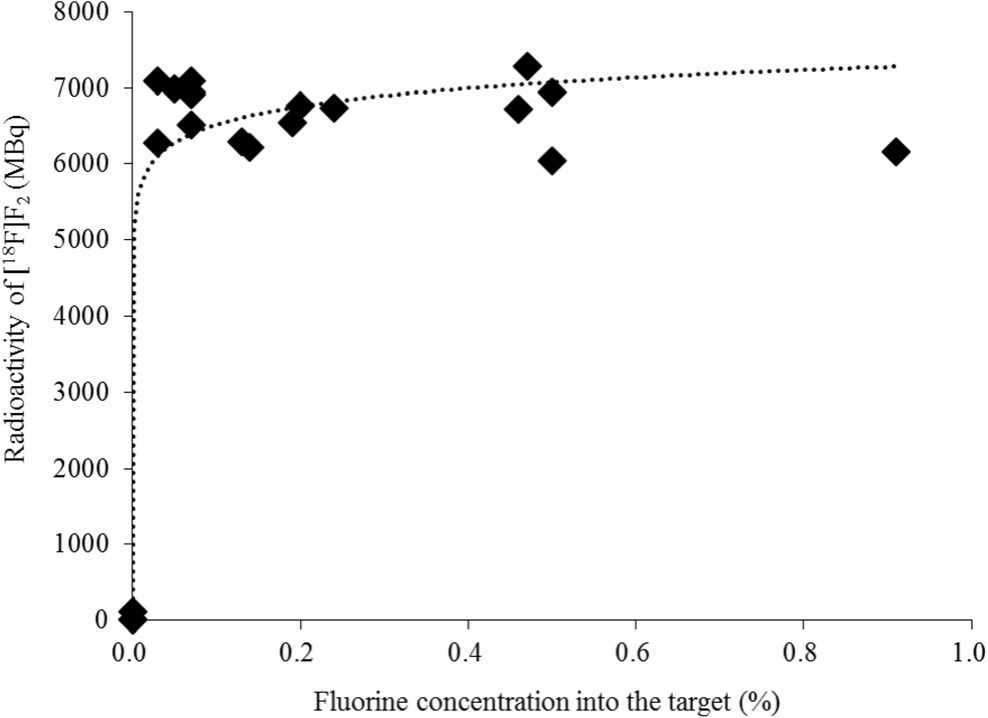
Radioactivity of [^18^F]F_2_ at each fluorine concentration in the target (0–0.9%); radioactivity (decay-correct to EOB) of [^18^F]F_2_ after second irradiation at 17 μA for 10 min

Figure 4 depicts the relationship between the radioactivity of [^18^F]F_2_ and the concentration of the F_2_ added in the argon gas. When only argon gas (without F_2_ gas) was used, a negligible amount of [^18^F]F_2_ was collected. However, the radioactivity of the [^18^F]F_2_ was constant when the concentration of the added F_2_ gas was increased up to 0.9%.

In the three-time synthesis of the [^18^F]FBPA solution, the mean amounts of [^18^F]F_2_ and [^18^F]CH_3_COOF were 55.0 ± 3.3 GBq and 21.6 ± 1.4 GBq, respectively, at the end of bombardment (EOB). The calculated conversion yield of [^18^F]F_2_ to [^18^F]CH_3_COOF was 39.2 ± 0.3%. The radioactivity of the [^18^F]FBPA was 4.72 ± 0.34 GBq at the end of synthesis (EOS), and the decay-corrected radiochemical yield of [^18^F]FBPA, based on [^18^F]F_2_, was 12.2 ± 0.1%. The time required for the synthesis of [^18^F]FBPA was 56.0 ± 2.0 min.

**Fig. 5:**
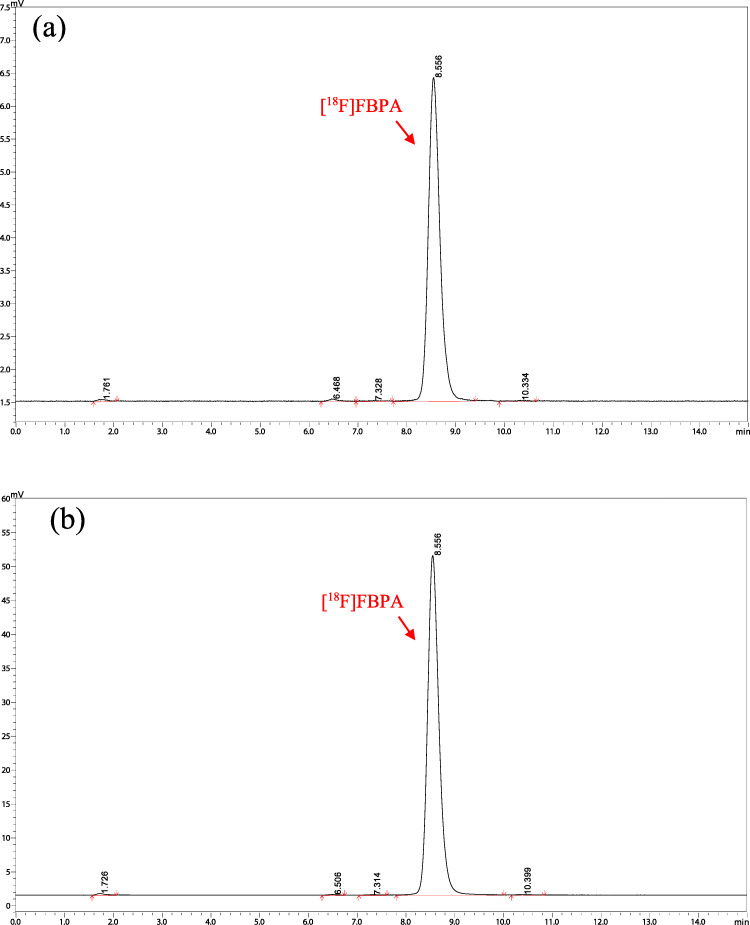
Radio-chromatograms of [^18^F]FBPA with analysis HPLC: **a** typical radio-chromatogram of [^18^F]FBPA solution at EOS; **b** typical radio-chromatogram of [^18^F]FBPA solution after 6 h of EOS

**Fig. 6:**
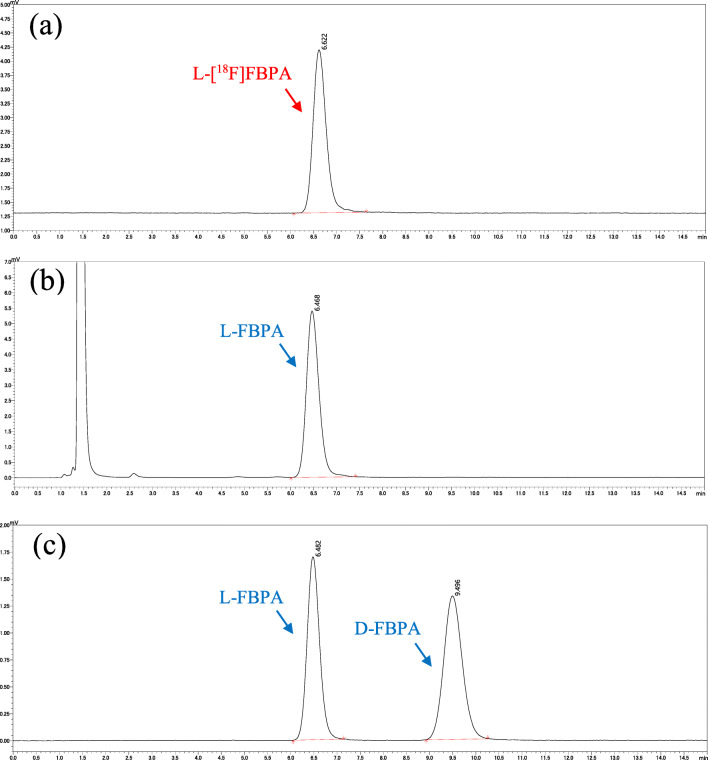
Chromatograms of [^18^F]FBPA with Chiral HPLC: **a** typical radio-chromatogram of [^18^F]FBPA solution; **b** typical UV-chromatogram of [^18^F]FBPA solution; **c** typical UV-chromatogram of the d-FBPA and l-FBPA reference standard mixed solution. Final concentration of the reference standard mixed solution was 0.25 mg/mL

The quality profiles of the [^18^F]FBPA solutions are summarized in Table 1. All the solutions satisfied the required criteria. The radiochemical purity was 99.3 ± 0.1% at EOS and 98.6 ± 0.2% after 6 h of EOS (Figure 5). The non-radioactive FBPA content was 0.3–0.4 mg/mL, and its molar activity was 231 ± 22 GBq/mmol. The ethanol, acetic acid and TFA as the residual solvent were <15 ppm, 38 ± 15 ppm, and < 15 ppm, respectively. All other quality control parameters satisfied the specification criteria. In addition, the optical purity of the resulting [^18^F]FBPA solution with the same synthesis protocol was >99 % (Fig. 6).

**Table 1:** Results of [^18^F]FBPA solution synthesis and quality control.

Test items	Acceptance criteria	Lot no. 1	Lot no. 2	Lot no. 3
Volume (mL)	10 ± 5	13.4	13.8	13.5
Radioactivity (^*^EOS) (MBq)	> 185	4,380	4,710	5,060
Half-life (min)	105–115	108.5	109.1	109.7
Color	Clear and colorless	Clear and colorless	Clear and colorless	Clear and colorless
Particles	None	None	None	None
Endotoxin (EU/mL)	< 0.25	< 0.025	< 0.025	< 0.025
Sterility	Sterile	Sterile	Sterile	Sterile
pH	5.0–8.0	5.6	5.7	5.6
Radionuclidic identity	Exhibits the peak at 511 keV	Exhibits the peak at 511 keV	Exhibits the peak at 511 keV	Exhibits the peak at 511 keV
Radionuclidic purity	Exhibits no peak except 511 keV and 1022 keV	Exhibits no peak except 511 keV and 1022 keV	Exhibits no peak except 511 keV and 1022 keV	Exhibits no peak except 511 keV and 1022 keV
Radiochemical purity (EOS) (%)	> 95	99.4	99.3	99.2
Radiochemical purity (6 h after EOS) (%)	> 95	98.6	98.5	98.8
Ethanol (ppm)	< 3,333	7	12	< 5
Acetic acid (ppm)	< 3,333	39	23	52
TFA (ppm)	< 400	< 15	< 15	< 15
Administrable injection volume (mL)	maximum dose < 5 mg/dose	16.7 (full dosage) (0.3 mg/mL)	16.7 (full dosage) (0.3 mg/mL)	12.5 (0.4 mg/mL)
Molar activity (GBq/mmol)	-	230	253	210

^*^EOS end of synthesis

## Discussion

We developed a method for producing [^18^F]FBPA through the nuclear reaction of ^18^O(p, n)^18^F using the existing target, which does not require the use of a [^18^O_2_]O_2_ gas-recycling system for [^18^F]F_2_ production, and employs [^18^F]CH_3_COOF as the labeling agent. We successfully synthesized 4.72 ± 0.34 GBq of [^18^F]FBPA at EOS with a molar activity of 231 ± 22 GBq/mmol and a radiochemical purity of 99.3 ± 0.1% by optimizing the beam current, irradiation time, and concentration of F_2_ in argon for the Al target box. The production of [^18^F]F_2_ using [^18^O]O_2_ gas was performed only in facilities with dedicated systems including target box and recycle system, because [^18^O]O_2_ gas was mostly recycled. Therefore, a large amount of installation cost and continuous maintenance was required; thus, the production of [^18^F]F_2_ using ^18^O_2_ gas has been possible only in limited facilities. On the other hand, we have constructed that a new one-way gas filling system using [^18^]O_2_ gas, similar to the irradiation system for C-11 production, was possible to obtain stable radioactivity with high radiochemical purity by combining this system with [^18^F]CH_3_COOF. In addition, as an improvement of practicality, we have elucidated that by using a target box for [^11^C]CO_2_, the synthesis using [^18^F]F_2_ including [^18^F]FBPA can be performed for clinical use at many PET cyclotron facilities where proton beam can be irradiated.

In the method originally developed by Ishiwata et al., a radioactivity of 0.85 ± 0.20 GBq, a molar activity of 54 ± 7 GBq/mmol, and a radiochemical purity of 99.5 ± 0.4% (at EOS) were obtained for [^18^F]FBPA, based on data obtained from the daily production at Osaka University Hospital (*n* = 21) (unpublished data). The proposed method improved the radioactivity five-fold, the molar activity 4-fold, and obtains equivalent highly radiochemical purity of [^18^F]FBPA. According to the guidelines for [^18^F]FBPA studies issued by the Japanese Society of Nuclear Medicine (Version 1.0), the administration of 3.7–5.5 MBq/kg of [^18^F]FBPA is recommended for a single patient [20]. Considering an assumed patient body weight of 60 kg and an [^18^F]FBPA administration dose of 4.0 MBq/kg, with 30 min duration for whole-body PET/CT scanning per patient, a single synthesis of [^18^F]FBPA according to the synthesis method adopted in this study, allows the study of 7–8 patients using one PET/CT scanner. Furthermore, in this study, we employed 0.1% aqueous acetic acid, as reported by Ishiwata et al., as the eluent for HPLC [11]. However, when 1 mM phosphate-buffered saline (PBS), pH = 6.7, was used as an eluent for the same purification, which does not require the additional eluent-evaporation step, and affords a 32 min synthesis time for the final [^18^F]FBPA solution [12]. The application of the PBS eluent to the present study could further increase the radioactivity by 16% due to the reduced synthesis time.

The key bottleneck in synthesizing a large amount of [^18^F]FBPA was the lack of a suitable method for producing large amounts of [^18^F]F_2_. A number of focused studies have been reported on the irradiation methods, target box materials, and the constitution of the target gas required for [^18^F]F_2_ production [13, 14, 16, 17]. In their [^18^F]F_2_ production process with ^18^O(p, n)^18^F, Bishop et al. reported that approximately 13% of the generated [^18^F]F_2_ was wasted after the first irradiation and 87% remained in the target system [14]. After the second, third, and fourth irradiations, the amounts of [^18^F]F_2_ were 54%, 23%, and 10% of the total [^18^F]F_2_, respectively. In this study, 3% of [^18^F]F_2_ was collected after the first irradiation, such that 97% of the [^18^F]F_2_ remained in the target system before the second irradiation. After the second, third, and fourth irradiations, the collected amounts of [^18^F]F_2_ were 75%, 16%, and 6%, respectively. In both studies, the second irradiation process consistently provided the highest fraction of [^18^F]F_2_. The reason for the difference observed between Bishop’s (54%) and our study (75%) may be attributed to the difference in the passivation conditions for the pre-irradiation (concentration of F_2_ gas in argon; 100 μmol vs. 224 μmol, proton beam current; 10 μA vs. 17 μA, respectively) or contamination of F_2_ into [^18^O]O_2_ gas by recycling. In our system, the [^18^O]O_2_ gas used in the first irradiation was not recycled for the subsequent [^18^F]F_2_ production and was exhausted; therefore, the [^18^O]O_2_ gas used was always fresh. In contrast, in terms of cost concerns for single-use of [^18^O]O_2_ gas, we estimated that the [^18^F]FBPA could be produced 80 times with one [^18^O]O_2_ cylinder, which means more than 600 patients could undergo [^18^F]FBPA-PET examinations per cylinder. Although one [^18^O]O_2_ cylinder costs more than US$20,000, the cost of [^18^O]O_2_ per patient is estimated to be less than US$40, which is only twice the cost of [^18^O]H_2_O for [^18^F]FDG production.

In the second irradiation, the concentration of the F_2_ carrier gas was also important to the release of the adherent [^18^F]F_2_. Hess et al. reported that a reduction in the F_2_ carrier gas concentration was associated with a decrease in the [^18^F]F_2_ production and an increase in molar activity [18]. In this study, [^18^F]F_2_ was collected when more than 0.03% of F_2_ was added as a carrier gas. The amount of [^18^F]F_2_ obtained was constant and was in the range 6000–7000 MBq of [^18^F]FBPA when the F_2_ gas concentration was 0.03–0.9%. In this study, we used 0.05–0.08% of the F_2_ carrier gas to obtain the maximum amount of [^18^F]F_2_ with a high molar activity. In this high-volume target box, the amount of [^18^F]F_2_ positively correlated with the intensity of the proton beam and irradiation time, which was in good agreement with the results reported by Hess et al. [18].

For the [^18^F]FBPA synthesis, Mairinger et al. developed a method using the nuclear reaction of ^18^O(p, n)^18^F, by employing [^18^O_2_]O_2_ as the target gas for the recycling system for [^18^F]F_2_ production, with [^18^F]F_2_ as the labeling agent. In their study, the radioactivity, radiochemical yield based on [^18^F]F_2_, molar activity, and radiochemical purity (at EOS) of [^18^F]FBPA were 5.3 ± 1.2 GBq, 8.5 ± 2.0%, 257 ± 37 GBq/mmol, and 98 ± 1%, respectively. The total synthesis time was 72 ± 7 min. Compared with their results, the proposed method provides an equivalent amount and molar activity of [^18^F]FBPA, and a higher radiochemical yield and radiochemical purity in a shorter synthesis time.

**Fig. 7.**
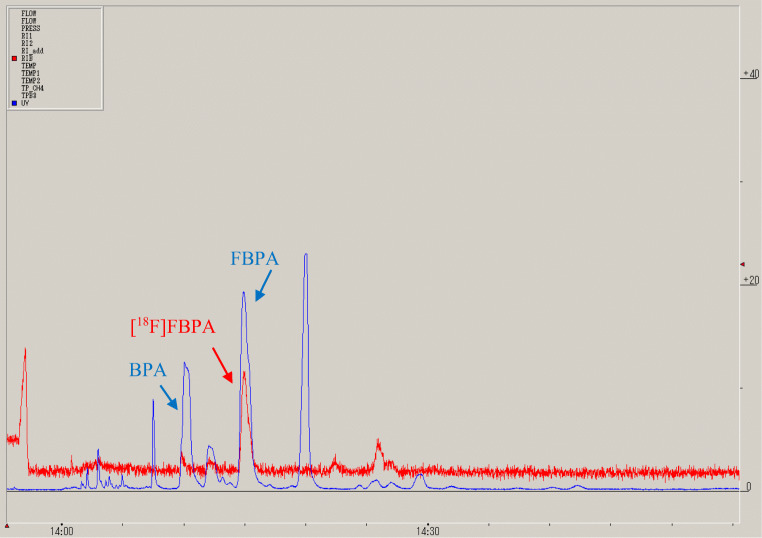
Typical separation HPLC chromatogram of [^18^F]FBPA solution; red line indicates the radioactivity peak while blue line indicates the UV (280 nm) peak

In this study, we selected [^18^F]CH_3_COOF as the labeling agent instead of [^18^F]F_2_, because of the higher radiochemical purity of [^18^F]FBPA as well as the higher ^18^F-labeling rate realized. Ishiwata et al. reported that the labeling rate of [^18^F]FBPA using a [^18^F]CH_3_COOF was approximately 50% whereas that using [^18^F]F_2_ was approximately 35 % [11]. This means that the production of radioactive byproducts in the [^18^F]FBPA produced using [^18^F]CH_3_COOF is decreased and could be almost separated from the radioactive byproducts (the separation HPLC chromatogram is presented in Fig. 7), whereas that produced with [^18^F]F_2_ contained detectable fractions of radioactive byproducts, such as the meta isomer due to insufficient separation [11, 12]. Most of the byproducts in the case of [^18^F]CH_3_COOF were three byproducts of BPA deboronation by electrophilic fluorination (2-[^18^F]fluorophenylalanine, 3-[^18^F]fluorophenylalanine; and 4-[^18^F]fluorophenylalanine) and this ratio was 25–35% in total [11]. However, their retention times were sufficiently longer than that of [^18^F]FBPA to allow easy separation, and the same chromatograms were obtained in our results. Furthermore, Ishiwata et al. reported that the radiochemical purity of [^18^F]FBPA synthesized with [^18^F]F_2_ was low when purified under the same HPLC conditions [12]. Similarly, our study showed that the same result was obtained (unpublished data). The higher radiochemical purity obtained in this study (99.3 ± 0.1%) than that obtained by using [^18^F]F_2_ as the labeling agent (98 ± 1%) [15] may be attributed to the differences in the variety and quantity of radioactive byproducts obtained using the two processes.

Furthermore, it has been reported that the synthesis of [^18^F]FBPA was performed by using [^18^F]F_2_ produced from [^18^F]fluoride [21–23]. This method has the advantage of providing high molar activity (0.9–1.5 GBq/μmol); however, it is not suitable for regular use because of the low labeling rate (3.4 % as calculated from [^18^F]fluoride) and the need for complex manufacturing systems and radioactivity detector for measurement of radio-gases [22].

In a [^18^F]FBPA synthesis, the radio-optical purity and its mass production were important, and our group had previously reported on the superiority of l-[^18^F]FBPA over d-[^18^F]FBPA as a cancer diagnostic agent [24]. Ishiwata et al. reported that >99.9% of [^18^F]FBPA with the original method was l-form [12], and we confirmed the same results for high radioactivity of [^18^F]FBPA synthesized from [^18^F]CH_3_COOF using the ^18^O(p, n)^18^F nuclear reaction. From this result, the use of [^18^F]FBPA may expand the available diagnostic cancer-seeking tracers with PET/CT beyond the examination before BNCT.

## Conclusion

We developed stabile and practical method for producing [^18^F]FBPA, which allowed the synthesis of a larger amount of [^18^F]FBPA than the original ^20^Ne(d, α)^18^F-based [^18^F]F_2_ method. Furthermore, the combination of [^18^O]O_2_ gas single-use system and [^18^F]CH_3_COOF as the labeling agent was more stable and practical compared with the method using [^18^O]O_2_ gas-recycling and [^18^F]F_2_ as the labeling agent. This synthesis process produced an equivalent amount of [^18^F]FBPA as the ^18^O(p, n)^18^F-based method and did not require any [^18^O]O_2_ gas-recycling.

## Data Availability

Contact the corresponding author for data requests.
